# Critique on “Epicardial Fat Thickness as a Marker of Coronary Artery Disease in Diabetics: A Single Center Study”

**DOI:** 10.1002/clc.70273

**Published:** 2026-03-05

**Authors:** Vishan Das, Rehab bint E Tahir, Safia Bibi, Hasnain Wajeeh Saqib

**Affiliations:** ^1^ Liaquat University of Medical & Health Sciences Jamshoro Sindh Pakistan; ^2^ HBS medical and dental college Islamabad Islamabad Pakistan; ^3^ Quetta Institute of Medical Sciences Quetta Pakistan; ^4^ Islamic International Medical College Riphah International University Rawalpindi Pakistan

**Keywords:** coronary artery calcium, coronary artery disease, diabetes mellitus, echocardiography, epicardial fat thickness, risk stratification

**Abbreviations**
AI: artificial intelligence
CAC: coronary artery calcium
CAD: coronary artery disease
EFT: epicardial fat thickness

To the Editor,

1

We read with great interest the article “Epicardial Fat Thickness as a Marker of Coronary Artery Disease in Diabetics: A Single Center Study” by Abdul Nadeem Akhter et al, in which the investigators examined the association of epicardial fat thickness (EFT) with coronary artery disease (CAD) among patients with diabetes [1]. A critical clinical question is addressed: Does echocardiographic EFT reliably stratify cardiometabolic risk in this population, and can it serve as a clinically actionable marker of CAD severity? The authors analyzed a large cohort (*n* = 2340) using uniform echocardiographic methodology and multivariable regression, reporting that higher EFT was significantly associated with increased CAD burden. Their findings provide important insights into the pathophysiological role of EFT in diabetic cardiovascular risk.

The authors' selection of a large sample size, consistent imaging protocol, and robust regression models is commendable. Their clear reporting of baseline characteristics and analytic approach makes the main point evident: EFT may represent a novel echocardiographic marker of cardiovascular risk in diabetes.

The immediate application of these findings to routine practice, however, is limited by several challenges. First, external validity requires greater emphasis. Recent studies demonstrate that population‐specific variations in EFT significantly influence cardiometabolic risk, thereby limiting the generalizability of findings across diverse ethnic and demographic groups [2]. Second, the dichotomous cut‐off (≥5 mm) offers analytical simplicity but risks misrepresenting a continuous biological variable such as EFT. This approach reduces statistical power and may obscure clinically relevant thresholds; modeling EFT as a continuous measure or with ROC‐derived cut points would provide a more nuanced understanding [3]. Third, the study does not assess whether EFT provides incremental prognostic value beyond established tools such as coronary artery calcium (CAC) scoring. Prior evidence shows that increased EFT is associated with higher CAC burden and greater mortality, raising the question of whether EFT independently predicts CAD severity or largely reflects established risk captured by CAC [4]. Finally, while the authors minimized inter‐operator variability by relying on a single echocardiographer, intra‐operator variability remains an inherent limitation. Advances in artificial intelligence (AI) assisted echocardiography now enable standardized, reproducible EFT measurement, reducing both intra‐ and inter‐operator error and offering greater precision than conventional techniques [5]. Importantly, despite these limitations, echocardiographic EFT measurement can be incorporated into routine cardiovascular evaluation, particularly in patients with diabetes, where it may aid early risk stratification and guide preventive management strategies.

Future research should (a) validate EFT thresholds across ethnically diverse cohorts, (b) adopt continuous or dynamic modeling approaches, (c) test incremental prognostic value over CAC and other established biomarkers, and (d) evaluate AI‐assisted echocardiography for reproducibility. These refinements, if confirmed, will help define the clinical role of EFT in cardiovascular risk stratification and its integration into patient care.

In summary, Abdul Nadeem Akhter et al offer valuable evidence that EFT is closely linked to CAD in diabetes. Their findings should stimulate further research into standardization, prognostic utility, and integration with advanced imaging techniques to establish EFT as a reliable clinical tool.

## Author Contributions

All Authors have equal contributions.

## Funding

The authors received no specific funding for this work.

## Ethics Statement

The authors have nothing to report.

## Consent

The authors have nothing to report.

## Conflicts of Interest

The authors declare no conflicts of interest.

## Data Availability

Data can be made available upon reasonable request.
